# *Pseudomonas* sp. as a Source of Medium Chain Length Polyhydroxyalkanoates for Controlled Drug Delivery: Perspective

**DOI:** 10.1155/2012/317828

**Published:** 2012-02-08

**Authors:** Sujatha Kabilan, Mahalakshmi Ayyasamy, Sridhar Jayavel, Gunasekaran Paramasamy

**Affiliations:** UGC-Networking Resource Centre in Biological Sciences, School of Biological Sciences, Madurai Kamaraj University, Madurai 625021, India

## Abstract

Controlled drug delivery technology represents one of the most rapidly advancing areas of science. They offer numerous advantages compared to conventional dosage forms including improved efficacy, reduced toxicity, improved patient compliance and convenience. Over the past several decades, many delivery tools or methods were developed such as viral vector, liposome-based delivery system, polymer-based delivery system, and intelligent delivery system. Recently, nonviral vectors, especially those based on biodegradable polymers, have been widely investigated as vectors. Unlike the other polymers tested, polyhydroxyalkanoates (PHAs) have been intensively investigated as a family of biodegradable and biocompatible materials for *in vivo* applications as implantable tissue engineering material as well as release vectors for various drugs. On the other hand, the direct use of these polyesters has been hampered by their hydrophobic character and some physical shortcomings, while its random copolymers fulfilled the expectation of biomedical researchers by exhibiting significant mechanical and thermal properties. This paper reviews the strategies adapted to make functional polymer to be utilized as delivery system.

## 1. Introduction

For the last decades, drug delivery systems have enormously increased their performances, moving from simple pills to sustained/controlled release and sophisticated programmable delivery systems. Currently, drug delivery has also become more specific from systemic to organ and cellular targeting [1]. In general, the action of a drug molecule is dependent on its inherent therapeutic activity and the efficiency with which it is delivered to the site of action. An increasing appreciation of the latter has led to the evolution and development of novel drug delivery systems (NDDSs) [2], whereas traditional delivery systems (TDSs) are characterized by immediate and uncontrolled drug release kinetics [3]. Accordingly, drug absorption is essentially controlled by the body's ability to assimilate the therapeutic molecule and thus, drug concentration in different body tissues, such as the blood, typically undergoes an abrupt increase followed by a similar decrease. As a consequence, increasing attention has been focused on drug delivery methods which continually delivers drugs for prolonged time periods and in a controlled fashion. The primary method of accomplishing this controlled release has been through incorporating the existing drugs into new drug delivery systems such as polymers. This novel approach considerably improves drug performance in terms of efficacy, safety and patient compliance. A large number of both natural and synthetic polymers have been studied for possible application in an outstanding range of extended/controlled release properties for a wide variety of dosage forms and processing methods. Among the polymer tested, two promising synthetic polymers which have been developed for biomedical applications are polyvinylpyrrolidone and polyethylene glycol acrylate-based hydrogels [3]. Both of them are biodegradable and form copolymers with natural macromolecules. On the other hand, natural polymers have the advantage of high biocompatibility and less immunogenicity [4]. Among the natural polymers studied a special mention has to be made to polyhydroxyalkanoates (PHAs). Other natural polymers are chitosan, alginate, starch, pectin, casein and cellulose derivatives. However, in recent years additional polymers designed primarily for medical applications have entered the arena of controlled release because of its biodegradability within the body; among them Polylactic acid (PLA), Polyglycolic acid (PGA), Poly (lactic-co-glycolic acid) (PLGA), Polycaprolactone (PCL), especially PHA (polyhydroxyalkanoate), and PHB (polyhydroxybutyrate) have attracted researcher's attention. Polyhydroxyalkanoates (PHAs) are bacterial polymers that are formed as naturally occurring storage polyesters by a wide range of microorganisms usually under unbalanced growth conditions. PHAs are composed of *β*-hydroxy fatty acids, where the R group changes from methyl to tridecyl. Poly (3-hydroxy butyrate) is the most investigated PHA [5]. Initially interests on PHAs were focused as replacements for petrochemical plastics such as polyethylene and polypropylene, due to their degradable nature (degradable to carbon dioxide and water through natural microbiological mineralization), but currently due to their biocompatibility, processability and degradability, PHAs have been investigated as matrices for drug delivery and tissue engineering applications [6]. Microorganisms are able to incorporate up to 60 different types of monomer into their storage polymer and a series of PHAs with different monomeric compositions (i.e., different physical and chemical properties (Table 1) can be produced [7]. Over the past years, PHAs, particularly PHB, have been used to develop devices including sutures, repair devices, repair patches, slings, cardiovascular patches, orthopedic pins, adhesion barriers, stents, guided tissue repair/regeneration devices, articular cartilage repair devices, nerve guides, tendon repair devices, bone marrow scaffolds, and wound dressings [8]. Furthermore, the *in vitro *and *in vivo *biocompatibility experiments demonstrated that PHB can be exploited for the purpose of encapsulation and controlled release of different drugs [8, 9]. Formulation performance can be optimized by variations in molecular weights and chemical substitutions. Each range has fundamentally different hydrophilicity, swelling and erosion characteristics which provide flexibility in controlling the release mechanisms.

## 2. Advantages of Using Natural Polymer

For the past decades, polymeric materials have been used for a variety of applications ranging from food industries, textile and biomedical industries. Biopolymeric materials may be utilized for the encapsulation, delivery of various functional food ingredients, drugs such as bioactive lipids, minerals, enzymes and peptides [11–13]. Most of the polymers initially used for drug delivery applications were hydrophobic and nondegradable in nature for example, poly(dimethylsiloxane) (PS), polyurethanes (PUs) and poly(ethylene-*co-*vinyl acetate) (EVA) [6]. Among the synthetic and natural polymers tested, biopolymers accumulated by microbes such as Polyhydroxyalkanoates (PHAs) and polyhydroxybutyrates (PHB) are attractive carrier matrices for drugs where the drugs can be released by bioerosion. At the same time, materials, which degrade or denature soon after processing, pose a significant threat for mass production and industrial usage leading to limited research interests beyond academia [12]. In order to make biopolymers having broad industrial/medical relevance, it would be better to form composite by incorporating with cross-linking agents. The modification of biopolymers with the addition of functional groups is a common yet elegant mechanism to create durable and industrially relevant biopolymers. Natural polymeric drug delivery systems have the following advantages over other controlled release formulations such as good biocompatibility, flexible drug release profile which could be adjusted through the cross-linking strategies, degradability of the by-products of the polymer and possibility of quick elimination by the excretory system to overcome accumulation in the body.

## 3. Biosynthesis, Structure, and Properties of PHA

**Figure 1:**
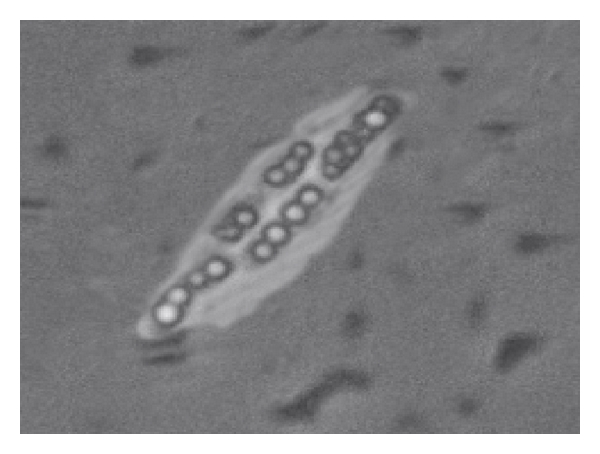
Phase Contrast Microscopic view of *Pseudomonas *sp. LDC-5 cells with accumulated PHA granules [24].

**Figure 2:**
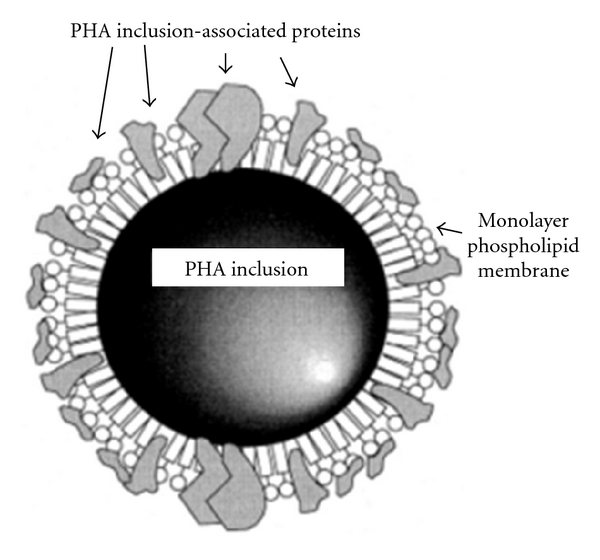
The structure of *in vivo *PHA inclusions and its association with specific proteins [7].

**Figure 3:**
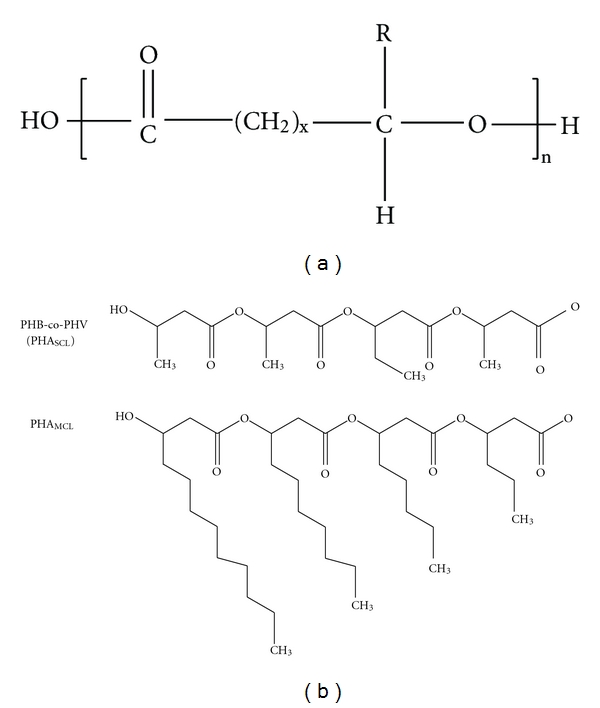
Chemical structure of PHA (a) and other classes (b) [10].

Polyhydroxyalkanoates (PHAs) are biological polyesters accumulated by microorganisms as energy reserve material in the form of intracellular granules. The layer of phasins (granule associated proteins) stabilizes the granules and prevents coalescence of granules in the cytoplasm (Figures 1 and 2). Decades of PHA research were dedicated to understand the production, its material properties and potential applications [14–17]. The mechanical property of PHA is dependent on the side chain length of hydroxyalkanoates. Based on that, PHAs were classified as SCL, MCL, and LCL-PHA consisting of 3~5, 6~16 and >16 carbon atoms respectively [18] (Figure 3). Unlike SCL-PHAs, MCL-PHAs have low levels of crystallinity and are more elastic [19, 20]. Recently, reports on PHA consisting of both SCL and MCL 3-hydroxyalkanoate (3HA) monomers have demonstrated a broader spectrum of application properties [7]. Though many Pseudomonads belonging to rRNA-DNA homology group I produce PHA polymers containing Medium-Length alkyl side chains (MCL-PHA), only a few wild-type bacteria such as *Pseudomonas *sp.61-3 [21], *Pseudomonas oleovorans *strain B-778 [22], and *Pseudomonas stutzeri *[12] were found to produce a mixture of PHB and MCL-PHA. Although some of these monomers have been found in PHA produced by bacteria in their natural environment, a larger fraction of monomers have been incorporated into PHA following growth of bacteria under laboratory conditions in media containing exotic sources of carbon. *Cupriavidus necator, Rhodospirillum rubrum *and *Pseudomonas pseudoflava* are known to accumulate copolyesters composed of SCL monomer units only, while *Pseudomonas oleovorans, Pseudomonas putida* and other *Pseudomonas* strains biosynthesize copolyesters principally composed of MCL monomer units. To a considerable extent, the substrate specificity of the PHA synthases determines the composition of the accumulated PHA. Biosynthesis of PHA is possible due to PHA synthases exhibiting extraordinarily broad substrate ranges [23]. Table 1 shows bacterial strains capable of producing P (3HA) polymers, the types of polyhydroxyalkanoate (PHA) synthases associated with those strains and the type of P (3HA) polymers produced by those bacteria.

The size, core composition and surface functionality can be highly controlled and provide a platform technology for the production of functionalized, biocompatible and biodegradable particles, which can be applied for drug delivery, diagnostics, bioseparation, protein immobilization and so forth [25–27]. Thus, the value of the polymers can be increased by controlling the polymer's microstructure. The physical properties of PHA homopolymers as well as co- and heteropolymers have been the subject of study in various laboratories all over the world. By controlling the monomer composition of PHA, polymer scientists have shown that the polymer's physical properties can be regulated to a great extent. Furthermore, it is also clear that the rate of degradation of PHA in various environments can be controlled by judiciously altering its monomer composition.

## 4. Polymer-Based Drug Delivery Systems

In general, drug delivery systems can be classified into liposomal, electromechanical and polymeric delivery systems. Though these systems are promising, the recent focus is on degradable polymeric matrices as drug carriers. The drugs are incorporated in a polymer and applied to facilitate the targeted drug delivery. The release rate depends on various parameters like nature of the polymer matrix, matrix geometry, properties of the drug, initial drug load and polymer-drug interactions [6]. Currently biopolymers are attractive drug carriers as these polymers need not be removed after their function is over. Poly (3-hydroxybutyrate) was the first homopolymer of PHAs which are intensively used for various applications. Earlier studies have reported the use of PHAs in implant biomedical devices and controlled drug-release carriers [8, 28, 29]. In addition to this, there have been reports indicating that PHB and PHBV copolymer of 3-hydroxybutyrate (3HB) and 3-hydroxyvalerate (3HV) can be used as extracellular controlled drug release matrices [30–32]. Reports by Xiong et al. [33] demonstrated that lipid soluble colorant rhodamine poly (3-hydroxybutyrate-co-3-hydroxyalkanoate) (RBITC) encapsulated in PHB and PHBHHX can be used for intracellular drug release. Above all, recent development in controlled drug delivery technology demands functionalized polymers for efficient performance. The following section will discuss the various strategies adopted to make functionalized polymers for drug delivery.

## 5. Improving the Biopolymer for Drug Delivery

Generally, drug delivery system to be designed in such a way is incapable of releasing its agent or agents (drugs) until it is placed in an appropriate biological environment, since biopolymers used in drug delivery are usually formed outside of the body and impregnated with drugs before placement the polymer plus drug complex in the body. In order to perform this, a wide range of cross-linking strategies can be used, including UV photopolymerization and various chemical cross-linking techniques. Such cross-linking methods are useful only if toxic reagents can be completely removed prior to implantation, which may be difficult to achieve without leaching loaded drug out of the polymer complex [34]. Though these approaches are advantageous, each method has its own advantages as well as limitations. In case of UV cross-linking due to their defined dimensionality and high elasticity excludes their extrusion through a needle. But these can be mitigated by making the complex into micro- or nanoparticles. Also, one has to consider the potential risks of exposure to UV and the cross-linking chemicals [34]. In general, the rate of drug release from a linear polymer matrix is inversely proportional to its viscosity.

## 6. Surface Functionalization of Biodegradable Polymers

Although many polymeric materials have been developed in the past decades to improve specific properties such as biocompatibility, degradability and drug delivery kinetics, there are also some limitations. In order to overcome this, copolymers with functional side groups which modify the surface with biologically active moieties may be useful [35–37]. Biomolecules such as fibronectin [38], collagen [39], insulin [40] and the epidermal growth factor [41] have often been introduced on polymer surfaces to enhance cell attachment or cell proliferation. The surface modification can produce controlled densities of hydroxyl groups on the surface and then these groups provide sites for the covalent attachment of specific biomaterials such as proteins or peptides [42]. Lee et al. [43] have performed modification of the biopolymers with various lengths of fluorocarbon (F-polyesters) end groups; this may improve the controllable biodegradability at initial stages by controlling the surface composition of fluorocarbon groups. Kang et al. [44, 45] emphasized the importance of plasma glow discharge technique in which partially ionized gas consisting of equal numbers of positive and negative charges and a different number of unionized neutral molecules is subjected to a DC or radio frequency (RF) potential. In order to introduce functional groups to polymeric surfaces (surface modification), glow discharge is widely used at reduced pressure. The characteristic glow of these plasmas is due to electronically excited species producing optical emission in the ultraviolet or visible regions of the spectrum and is characteristic of the composition of the glow discharge gas. Argon gives a bright blue colour and air or nitrogen gives a pink color that is due to excited nitrogen molecules.

## 7. Cross-Linking Strategies

In order to increase the stability, there is a growing trend towards the development of innovative biopolymeric material through the rational design of functional structures by cross-linking using various physical, chemical and enzymatic approaches depending on the specific characteristics of the biopolymers involved, rather than the use of the more traditional trial and error approach. These crosslinked polymers exhibit the required functional attributes, for example, optical properties, rheological properties, release characteristics, encapsulation properties and physicochemical stability [11].

## 8. Cross-Linking through Physical Methods

Polymer-polymer interaction without covalent bonding is known as physical cross-linking [46]. This includes ionic cross-linking, hydrogen bonding and hydrophobic bonding. Physical cross-linking can be achieved using a variety of environmental triggers such as pH, temperature, ionic strength and physiochemical interactions such as hydrophobic, charge condensation, hydrogen bonding and stereocomplexation [34]. These methods may be used for the induction of gelation processes among already formed discrete biopolymer particles.

## 9. Temperature

Temperature either strengthens or weakens the interactions of holding biopolymer molecules together, depending on the nature of the dominant forces involved. In general, hydrophobic forces are strengthened with increasing temperature, hydrogen bonding is weakened, and entropy effects are increased. Heating (heat-set-gelation) increases the hydrophobic driven association and cooling (cold-set-gelation) increases hydrogen bonding-driven association [47]. These temperature-associated reactions are either reversible or irreversible. Heat-set-gelation is used to cross-link globular proteins, such as milk, soy and egg as well as cross-link polysaccharides with some hydrophobic character. Cross-linking using heat-set-gelation of globular proteins tends to be irreversible. Once the aggregates are formed at higher temperatures, they remain intact when the system is cooled below the thermal denaturation temperature. In this reaction, the system is cooled below the thermal aggregation temperature as the molecules tend to dissociate [48]. Cold-set-gelation is stable at relatively low temperatures, but it tends to dissociate when heated above a critical temperature. This kind of gelation involves those biopolymers that are capable of forming helical regions that associate with each other through hydrogen bonding upon cooling. In case of mixed biopolymer systems that segregate, decreased temperatures can be utilized to favor separation, which in turn increases the biopolymer interaction within the dispersed phase [49]. Increased biopolymer interactions within these excluded volumes resulted into gel-like matrices; further heating may be utilized to further solidify these compact phases into particulates [50].

## 10. pH

In general, the response in different regions of the body is dependent on pH; the release of drugs in controlled fashion is possible by modifying the polymer cross-linking. Changes in pH and addition of mineral ions may be used to promote biopolymer association through alterations in electrostatic interactions. For example, calcium ions (Ca^2+^) are frequently used to cross-link anionic polysaccharides such as pectins, alginates, or carrageenans [51]. Potassium ions (K^+^) are used to cross-link anionic carrageenans through the formation of an “egg-box” structure [51]. Addition of mineral ion may be used to cross-link biopolymer particles in various ways [52]. An alternative method is to utilize slow-releasing salt devices to initiate cross-linking for controlled release of drug.

## 11. Chemical Cross-Linking

Introduction of a covalent linkage between polymer functional group is known as chemical cross-linking. The chemical agents may act as a bridge between similar or dissimilar amino acids [53]. They may also be used to directly bond 2 amino acids together. Functional groups capable of chemical bonds include amines, thiols, hydroxyl groups and phenyl rings. These chemical cross-linking strategies are widely used by researchers all over the world in comparison to physical cross-linking of functional groups in biopolymer.

## 12. Amphiphilic PHAs

PHAs are promising materials for biomedical applications in tissue engineering and drug delivery system because of their properties such as natural, renewable, biodegradable and biocompatible thermoplastics. The key to biocompatibility of biomedical implantable materials is to render their surface in a way that minimizes hydrophobic interaction with the surrounding tissue. Therefore, hydrophilic groups need to be introduced into the PHAs in order to obtain amphiphilic polymer. Amphiphilic polymers can be synthesized by introducing hydrophilic groups such as hydroxyl, carboxyl, amine, glycol and hydrophilic polymers such as PEG, poly(vinyl alcohol), polyacryl amide, poly acrylic acids, hydroxy ethyl methacrylate, poly vinyl pyridine and poly vinyl pyrrolidone to a hydrophobic moiety by means of functionalization and grafting [54]. Among the hydrophilic groups, PEG is a polyether known for its exceptional blood and tissue compatibility. It is used extensively as biomaterial in a variety of drug delivery vehicles and is also under investigation as surface coating for biomedical implants. PEG, when dissolved in water, has a low interfacial free energy and exhibits rapid chain motion and its large excluded volume leads to steric repulsion of approaching molecules [55].

## 13. Transesterification

Under certain conditions, esters and amides are capable of undergoing interchange reactions in aqueous solutions. Transesterification is carried out either in melt or in solution. Transesterification reactions in the melt between poly(ethylene glycol), mPEG, and PHB yield diblock amphiphilic copolymer with a dramatic decrease in molecular weight [56]. Genipin is a naturally occurring heterocyclic compound derived from *Genipa americana *that is able to form physical links between biopolymers containing amines. Mi et al. [57] reported that the primary amine groups located on biopolymers attack at either the *α*- or *β*-carbon of the genipin ester. During the attachment at the *α*-carbon that forms a simple amide linkage, attack at the *β*-carbon causes ring cleavage and further cross-linking capability. So far the genipin crosslinked biopolymers are chitosan, BSA, soy protein and gelatin [58]. Furthermore, the natural compound genipin is considered to be less cytotoxic than other cross-linking agents such as glutaraldehyde [57].

## 14. Maillard Reactions

Biopolymers are capable of loading both hydrophilic and hydrophobic drugs; in particular, modified polymers without synthetic chemical reagents are obviously desirable for biomedical applications. Several groups have studied the polymer fabrications for controlled release applications. One such fabrication is Maillard reaction; Maillard reaction is a natural, nontoxic reaction that occurs during the processing, cooking and storage of foods. As per, Oliver et al. [59], Maillard reaction is chemical linking of aldehydes and amines through a well-established oxidation-reduction pathway. High pH values favored these imido- and redox reactions. Maillarad conjugates have been used as emulsifiers and gelling agents. For example, the conjugation of hen egg lysozyme with dextran, galactomannan, or xyloglucan is effective in improving the emulsifying activity of the protein and it has been shown that the conjugated lysozyme has new antimicrobial characteristics [60, 61]. Rich and Foegeding (2000) [62] demonstrated that these Maillard reactions are useful to cross-link protein components with mono- and disaccharides. Recent, reports by Elzoghby et al. [63] have shown the significance of casein-based formulations as promising controlled release drug delivery systems. Casein, the major milk protein, is a good candidate for conventional and novel drug delivery systems for its property with high tensile strength. This Maillard reaction could also be used to cross-link proteins and polysaccharides within the biopolymer particles formed by spray-or freeze-drying. After drying, the system can then be subjected to dry-heating to promote the Maillard reaction.

## 15. Aldehyde Reactions

Glutaraldehyde and formaldehyde can be used to chemically cross-link protein particulate system. These may form compounds such as acetals, cyanohydrins, and oximes [64]. At the same time, studies by [65] showed that even 3 ppm of nonfood grade aldehydes exhibits cytotoxic effects to human fibroblasts. As a result, attempts were put forth to identify a number of food-grade alternatives to replace the potentially toxic compounds.

## 16. Quaternization and Sulfonation of the PHAs

Addition of the chlorine and bromine into the double bond is quantitative and halogenated PHAs can be easily obtained by this approach [66]. Chlorination can be done by either the addition to double bonds of the unsaturated PHA obtained from soybean oil (PHA-Sy) or substitution reactions with saturated hydrocarbon groups [67, 68]. Chlorination provides polyester with hard, brittle and crystalline physical properties depending on the chlorine content and also glass transition temperature has been shifted from *−*40°C to +2°C [68]. For further functionalization, quaternization reactions of the chlorinated PHA with triethylamine (or triethanol amine) can be performed.

## 17. Enzymatic Methods

Apart from physical and chemical cross-linking, enzymes can also be utilized to catalyze specific cross-linking reactions between polymer. They are particularly useful in applications where alternative methods might cause damage to some encapsulated component. At the same time, sulfhydryl or phenolic residues can be oxidatively cross-linked using high levels of gaseous oxygen, but this would be deleterious to high-value lipids or phenolics [69]. In this case, usage of specific enzymes is a very good alternative. Recent reports on laccases (benzenediol: oxygen oxidoreductases; EC 1.10.3.2.) which are glycosylated polyphenol oxidases containing four copper ions per molecule, produced by white rot fungi in large amounts, have widespread applications such as effluent decolouration pulp bleaching and removal of phenolics from wines, organic synthesis, biosensors and synthesis of complex medical compounds, among others. Laccases are also able to cross-link biopolymers containing phenolic acids like ferulated arabinoxylans [70–72]. In particular, recently, enzymatic cross-linking and grafting of specific substances to the biopolymers can be exploited in food and nonfood applications allowing for generation of novel biomaterials [73]. De Jong and Koppelman [74] have reported the importance of transglutaminase which is commonly produced from bacterial sources that can be used as cross-linking agents for various types of proteins. This enzyme functions by a nonoxidative transamidation between glutamine and lysine, whether intra- or intermolecularly. Transglutaminase has been used to produce crosslinked protein films from gelatin [75], egg-white [76], gluten [77], soybean and whey proteins [78]. The extent of cross-linking can be controlled by changes in pH, enzyme inhibitors, or heating [74].

## 18. Grafting

Some of the naturally occurring polymers such as polyhydroxyalkanoate, alginate and chitosan find increasing application in biomedical research due to their biocompatible, biodegradable and nontoxic properties. However, to overcome the limitations posed by these polymers such as low moisture resistance, poor processability and incompatibility with some hydrophobic functional groups, the effective modification method grafting is adopted. This method is used to prepare multifunctional materials with improved chemical, physical and mechanical properties. Chitosan, sugar, PLA, gelatin, and PEG-mediated grafting are discussed in several studies [79–82]. Among these, glycopolymers are emerging as a novel class of neoglycoconjugates useful for biological studies and they are prepared by either copolymerization or grafting methods. One another, recently noted hydrophobic polymer with biodegradable ketal linkages in its backbone has an advantage over current biodegradable polymers for drug delivery is Polyketals. Polyketals do not release inflammatory byproducts compared to existing polymers [82].

## 19. Conclusion

Presently, novel polymeric materials have revolutionized the polymer applications in various fields including pharmaceutical, food and agricultural applications, pesticides, cosmetics, and household products. Particularly, in the pharmaceutical field, in addition to the importance of polymers, an understanding of the physiological barriers in the human body is also critical to develop appropriate controlled release systems. The skin, the gastrointestinal tract, the nose and the eye are of particular importance. In the immediate future, one of the dominant factors to be expected from human endeavor is environmental friendliness. Along this line, serious efforts are mounted to the developments of biopolymers with appropriate properties and processability, the so-called “green” polymers, that contrast to the conventional petrochemically originated polymers. In the future, custom-made prominent MCL-PHA synthases generated through *Pseudomonas* enzyme evolution will be utilized extensively to create high-performance P (3HA)s in various organisms from renewable carbon sources or through improved *in vitro* systems.

## Figures and Tables

**Table 1:** Classes of PHA synthases and varieties of P (3HA)s (adapted from Rehm 2007) [10].

Substrate specificity	Class of PHA synthase	Subunit(s) (PHA synthase subunit)	Microorganism	Polymers produced^a^
SCL-3HA-CoA (C_3_–C_5_)	I	PhaC	*Ralstonia eutropha *	P (3HB), P (3HB-*co-*3HV)
	III	PhaC, PhaE	*Allochromatium *	
	IV	PhaC,PhaR	*vinosum * *Bacillus megaterium*	

MCL-3HA-CoA (C_6_–C_14_)	II	PhaC	*Pseudomonas *	P (3HA)
			*oleovorans *	
			*Pseudomonas putida *	
			*Pseudomonas aeruginosa*	

SCL-MCL-	I	PhaC	*Aeromonas caviae *	P (3HB-*co-*3HA)
P (3HA)-CoA (C_3_–C_14_)	II	PhaC	FA440	
			*Pseudomonas *sp.61-3	

**^a^**P (3HB), poly-3-hydroxybutyrate, P (3HB-*co-*3HV), poly-3-hydroxybutyrate-*co-*3-hydroxyvalerate, P (3HA), poly-3-hydroxyalkanoate, P (3HB-*co-*3HA), poly-3-hydroxybutyrate-*co-*3-hydroxyalkanoate.
